# We Need to Rethink on Medical Education for Pandemic Preparedness: Lessons Learnt From COVID-19

**DOI:** 10.4274/balkanmedj.galenos.2020.2020.4.002

**Published:** 2020-06-01

**Authors:** Dilek Aslan, İskender Sayek

**Affiliations:** 1 Department of Public Health, Hacettepe University School of Medicine, Ankara, Turkey; 2 Department of General Surgery, Hacettepe University School of Medicine, Ankara, Turkey

Novel coronavirus disease (COVID-19) is a pandemic and the efficiency of public health measures is being questioned, as the vaccine and specific treatment have not been found yet. Globalization and its impact on the determinants of health deepen the inequalities and this makes the situation more difficult to overcome. Questioning the missing points is not rare and the need for holistic and systematic approach is being emphasized frequently (1).

In the current picture, clinicians are playing the key role in the struggle with COVID-19 at the hospitals. This is somehow normal as COVID-19 with its many unknowns may be lethal and needs very much curative services. However, the disease is spreading mainly outside the hospital and controlling it in the community will be more efficient. Detecting the sick people, implementation of the basic steps like quarantine, isolation and segregation, improvement of personal preventive methods like social distancing, hygiene, face mask use are all the parameters of the success of the control at the community level.

Lack of comprehensive community-based programs about COVID-19 drew the discussion to the social, economic, cultural, and medical aspects extending to medical education. Most likely, there is a mismatch between the evidence-based science and the society responses to the scientific content. The national medical authorities also are not fully effective in convincing the people about the recommended measures. Such difficulties also question the content and the methods of medical education. Criticism of medicine has started since the COVID-19 pandemic based on all these issues (2).

At this point, the question “Can today’s medical education content really respond to the pandemic(s)?” is open for discussion. Before giving an “exact” answer, let us try to discuss how pandemic situations can affect societies.

Pandemic is one of the most complicated issues of humanity as experiences from the past have shown many dramatic casualties. In the previous centuries, cholera, black death, Spanish flue, and many others have affected the population health and caused high mortality and morbidity. At the mean time today, the world is familiar with epidemics like SARS, MERS-CoV, etc. and the recent one “COVID-19 pandemic”. COVID-19 does not have specific curative and vaccination options and such obscurity makes the situation more worrying among both medical staff and the community. Despite all the challenges, health professionals including doctors are working hard and are trying to do their best mostly ignoring their own health. COVID-19 is spreading fast, and health systems may be blocked as the disease becomes more severe and the need for intensive care increases. To flatten the curve and to mitigate the influence of the epidemic, prevention of the spread of the disease and detecting the cases at earlier phases are more vital in the struggle. In brief, community-based work should be planned and continuously conducted.

At this point, let us return to the question “Can today’s medical education approach really respond to the pandemic(s)?” Unfortunately, the answer is not “yes” depending on the experiences gained during COVID-19. Medical educators should think reorganizing the content for pandemic preparedness keeping. Highlighted points can be covered in the curriculum:

1. Pandemic simulations

2. Governance of pandemic, epidemiology

3. Concepts of social accountability, transparency, etc.

4. Ethical perspectives and dilemmas

5. Intra, inter, multi and trans-disciplinary approaches

6. Community based models

7. Community oriented models

In this context community oriented and community based medical education and social accountability of medical schools are the concepts which we need to understand better and implement them in all stages of medical education. Community orientation and social accountability indicates to relevance of objectives to meet the community health needs and then reflecting the content of the curriculum these objectives (3,4). Community-based medical education is the delivery of medical education in a specific social context. Learners become a part of social and medical communities where their learning occurs. Briefly, learning occurs in the community (5). If the medical schools use such approaches, implement the principles of community-based education and social accountability (6), the medical students will be much well prepared for the pandemics, working in the community and will respond to the needs of the society. Such preparation is expected to contribute to struggle with the future possible threats in a more realistic manner.
